# Short-Interval Mifepristone-Misoprostol Versus Misoprostol Alone for Second-Trimester Abortion: A Retrospective Observational Study

**DOI:** 10.7759/cureus.103544

**Published:** 2026-02-13

**Authors:** Ala Aiob, Dina Gumin, Raneen Abu Shqara, Inshirah Sgayer, Lior Lowenstein, Avishalom Sharon

**Affiliations:** 1 Obstetrics and Gynecology, Bar Ilan University Faculty of Medicine, Safed, ISR; 2 Obstetrics and Gynecology, Galilee Medical Center, Nahariya, ISR

**Keywords:** medical abortion, mifepristone-misoprostol regimen, missed abortion, second-trimester abortion, short-interval regimen

## Abstract

Introduction

The aim of this study was to evaluate the efficacy and safety of a short-interval (12-24 hours) mifepristone-misoprostol regimen compared with a misoprostol-only protocol for medical management of second-trimester fetal demise.

Material and methods

A retrospective observational study was conducted on women diagnosed with second-trimester fetal demise (13-23.6 weeks of gestation) who underwent medical uterine evacuation at a tertiary medical center. Patients were managed according to departmental protocols in effect during specific time periods and received either a mifepristone (600 mg) and misoprostol combination regimen (study group) or misoprostol alone (control group). The primary outcome was successful abortion, defined as fetal expulsion following the completion of the predefined misoprostol course without the need for additional misoprostol cycles or alternative interventions. Secondary outcomes included the induction-to-expulsion interval, predefined complications, and analgesic requirements. Multivariable logistic regression analysis was performed to identify factors associated with successful abortion, including age, gravidity, gestational age, and treatment protocol.

Results

A total of 178 women were included, of whom 118 received the mifepristone-misoprostol regimen and 60 received misoprostol alone. The success rate was significantly higher in the combination group compared with the misoprostol-only group (80.5% vs. 48.3%, p < 0.001). The overall complication rate, including profuse bleeding, infection, and need for additional intervention, was significantly lower in the combination group (1.7% vs. 10.0%, p = 0.031). No statistically significant difference was observed in the induction-to-expulsion interval between groups. Analgesic requirements were significantly lower in the combination group. Multivariable analysis demonstrated that use of mifepristone and increasing parity were independently associated with higher odds of successful abortion.

Conclusion

In women with second-trimester fetal demise, a short-interval mifepristone-misoprostol regimen is associated with significantly higher success rates and fewer complications compared to misoprostol alone. These findings support the effectiveness and safety of short-interval combination therapy in this clinical setting.

## Introduction

Second-trimester pregnancy termination may be necessary for various reasons, including unwanted pregnancy, limited access to early abortion services, maternal medical comorbidities, fetal anomalies, or intrauterine fetal death [1-4]. Both surgical and medical options are available, with the choice depending on patient preference, provider expertise, and the availability of required medications and instruments [5,6]. Hemodynamically unstable patients are generally not suitable candidates for medical induction and need alternative management approaches.

Misoprostol, a prostaglandin E1 analog, is the preferred agent for induction due to its effectiveness, cost-effectiveness, and ease of use [7]. However, the most effective medical regimen for second-trimester abortion involves combining mifepristone, a competitive progesterone receptor antagonist, with subsequent doses of misoprostol [7]. Mifepristone helps ripen the cervix and increases the myometrium's sensitivity to prostaglandins, thereby enhancing uterine contractions and significantly shortening induction time compared to misoprostol alone [8-10]. Therefore, current national and international guidelines recommend a 24-48-hour interval between mifepristone and misoprostol [7,11-15].

Several observational studies have explored varying mifepristone-to-misoprostol intervals, including ≤12 hours, 12-24 hours, and >24 hours, assessing their impact on total abortion duration [2,16]. Notably, induction times were clinically comparable across different regimens, and shorter intervals did not compromise safety or efficacy [2,8]. A systematic review concluded that shortening the mifepristone-misoprostol interval does not adversely affect the safety or effectiveness of second-trimester medical abortion and may better accommodate patient and provider preferences [8]. Most contemporary studies evaluating short-interval regimens have used a 200 mg dose of mifepristone followed by misoprostol. Reducing the total abortion duration may improve patient experience, shorten hospitalization, enhance clinical workflow flexibility, and support patient-centered care and shared decision-making [2].

This retrospective observational study evaluates the effectiveness and safety of inducing second-trimester fetal demise using a short-interval (12-24 hours) combination regimen of mifepristone (600 mg) and misoprostol, compared with a misoprostol-only protocol. By focusing on a higher mifepristone dose and a short administration interval, this study aims to address a clinically relevant gap in the existing literature and to clarify the additive value of this approach in contemporary practice.

## Materials and methods

This retrospective observational study was conducted at Galilee Medical Center, a tertiary referral hospital, and was approved by the Galilee Medical Center Institutional Review Board (IRB No. 0168-18NHR). Informed consent was waived due to the retrospective study design. Women diagnosed with second-trimester intrauterine fetal demise between 13+0 and 23+6 weeks of gestation who underwent medical uterine evacuation were eligible for inclusion. Gestational age was determined based on a reliable last menstrual period and confirmed by first-trimester crown-rump length measurement when available. Intrauterine fetal demise was defined as the absence of fetal cardiac activity confirmed by ultrasound examination, and in all cases, termination of pregnancy was initiated shortly after diagnosis according to institutional practice. Patients were excluded if they were hemodynamically unstable, had clinical or laboratory evidence of intrauterine infection, presented with an ongoing or incomplete abortion, or had spontaneous uterine contractions before initiation of medical treatment, as such patients are not candidates for medical induction.

Patients were identified through the hospital’s computerized medical records system. The extracted clinical data included demographic characteristics, obstetric history, gestational age, ultrasound findings, hemoglobin levels before and after the procedure, analgesic use, treatment protocol, time intervals, and clinical outcomes. Patients were managed according to standardized departmental protocols that varied by predefined time periods rather than individual physician preference. The control group received a misoprostol-only regimen consisting of an initial vaginal loading dose of 800 μg, followed by buccal misoprostol 400 μg every three hours for up to three additional doses. The study group received oral mifepristone 600 mg, followed 12-24 hours later by buccal misoprostol 400 μg every three hours for up to three doses; the 600 mg dose of mifepristone represented the standard institutional protocol during the study period. If fetal expulsion did not occur after completion of the predefined misoprostol course, further management options included a repeat misoprostol course, intrauterine balloon catheter placement, intravenous oxytocin infusion, or surgical intervention, according to institutional guidelines and clinical judgment. A misoprostol course was defined as the initial loading dose followed by up to three subsequent doses.

The primary outcome was successful abortion, defined as complete fetal expulsion following completion of the predefined misoprostol course without the need for additional misoprostol cycles or alternative abortion modalities. Treatment success or failure was assessed after completion of the protocol according to institutional guidelines. Secondary outcomes included the induction-to-expulsion interval, analgesic requirements, and predefined complications. The induction-to-expulsion interval was defined as the time from administration of the first dose of misoprostol to fetal expulsion; in cases in which expulsion occurred before administration of the fourth misoprostol dose, the interval was recorded as a negative value, reflecting expulsion before completion of the planned dosing schedule. Analgesic treatment followed a stepwise institutional protocol, with initial use of non-opioid analgesics and escalation to intravenous opioids based on clinical need and patient-reported pain, and total analgesic consumption was recorded.

Following fetal expulsion, uterine evacuation was assessed clinically and by ultrasound. Surgical curettage under general anesthesia was performed selectively in cases of suspected retained placental tissue and was not routinely performed in all patients. Rh-negative patients received 300 μg of anti-D immunoglobulin according to standard practice. Complications were predefined and included profuse bleeding, defined as a hemoglobin decline greater than 25% from baseline, fever ≥38°C, clinical infection, need for blood transfusion, intrauterine balloon catheter placement, oxytocin infusion, or surgical intervention; complications occurring within 24 hours of uterine evacuation were recorded.

Statistical analysis

Categorical data were described using frequencies and percentages. Continuous variables with normal distributions were presented as means ± standard deviations. Median values and ranges were used to describe variables that did not follow a normal distribution. Categorical variables were compared between the groups with the chi-square test or Fisher's exact test (when expectancy was <5). Continuous variables were compared between groups using the Mann-Whitney U test or the independent t-test, depending on whether the data were normally distributed. The distribution shape was determined mainly by a histogram. p < 0.05 was considered significant. Multivariable linear regression models were employed to examine the correlation between the success of the abortion process and independent variables, including age, gravidity, and protocol type. Statistical analysis was conducted using IBM SPSS Statistics software, version 25.0 (IBM Corp., Armonk, NY)

## Results

A total of 178 women met the inclusion criteria and were included in the analysis. Of these, 118 women were managed with the short-interval mifepristone-misoprostol regimen (study group), and 60 women received misoprostol alone (control group). The mean age of the study population was 30.3 years (17-47), the mean gravidity was 3.1 (1-8), and the mean gestational age at termination was 17.2 ± 2.6 weeks (range: 13.0-23.6 weeks). Baseline demographic and obstetric characteristics were comparable between the two groups, with no statistically significant differences in age, gestational age, gravidity, parity, or rates of medical comorbidities (Table 1).

**Table 1:** Baseline characteristics, clinical outcomes, and complications of patients undergoing second-trimester abortion with misoprostol alone vs. mifepristone-misoprostol Data are presented as mean ± SD, median (range), or number (%), as appropriate. Categorical variables were compared using the chi-square test. Continuous variables were compared using the t-test for normally distributed data and the Mann-Whitney U test for non-normally distributed data (reported as Z-values). A p-value < 0.05 was considered statistically significant. *Chi-square test; #Mann-Whitney U test. C1, first dose of misoprostol; C4, fourth dose of misoprostol; HGB, hemoglobin; IUI, intrauterine insemination; IVF, in vitro fertilization; SD, standard deviation.

	Misoprostol (N = 60)	Mifepristone-misoprostol (N = 118)	p-value	V-value
Age, mean (±SD)	30.5 (±6.7)	30.1 (±7.0)	N/S	
Parity, mean (±SD)	3.2 (±1.7)	2.9 (±1.6)	N/S	
Hypertension, n (%)	0	2 (1.7%)	N/S	
Diabetes mellitus, n (%)	1 (1.7%)	1 (0.8%)	N/S	
Hypothyroidism, n (%)	1 (1.7%)	3 (2.5%)	N/S	
Thalassemia, n (%)	0	3 (2.5%)	N/S	
Asthma, n (%)	0	2 (1.7%)	N/S	
Gestational age, mean (±SD)	17.0 (±2.4)	17.3 (±2.7)	N/S	
IUI, n (%)	0	1 (0.8%)	N/S	
IVF, n (%)	1 (1.7%)	1 (0.8%)	N/S	
Twins, n (%)	1 (1.7%)	6 (5.1%)	N/S	
Complication, n (%)	6 (10%)	2 (1.7%)	0.031^*^	χ² = 4.64
Perfused bleeding, n (%)	5 (8.3%)	1 (0.8%)	0.028^*^	χ² = 4.83
Fever, n (%)	1 (1.7%)	1 (0.8%)	0.028^*^	χ² = 7.14
C1 to abortion (hours), median (range), mean (±SD)	10 (1.9-24.5), 10.7 (±5.1)	8.5 (1.4-26.2), 9.7 (±5.1)	0.158^#^	Z = −1.41
C4 to abortion (hours), median (range), mean (±SD)	4 (−4.0-18.5), 4.6 (±4.9)	2.5 (−3.6-20.7), 4.0 (±5.2)	0.276^#^	Z = −1.10
Delta HGB (%), mean (±SD)	−11 (±10)	−8 (±9)	0.045^#^	t = −2.00
Course 2 of misoprostol, n (%)	26 (43.3%)	16 (13.6%)	0.001^*^	χ² = 19.56
Course 3 of misoprostol or other abortion modality, n (%)	5 (8.3%)	3 (2.5%)	0.078^*^	χ² = 3.10

**Figure 1:**
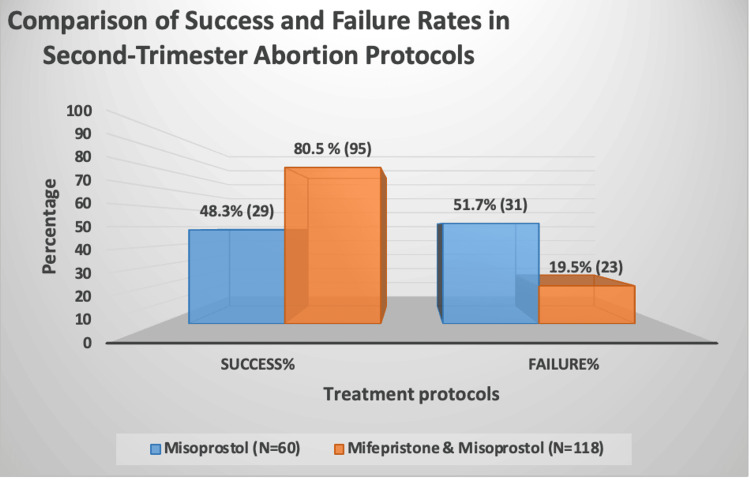
Comparison of success and failure rates between second-trimester abortion protocols Data are presented as n (%). Groups were compared using the chi-square test. A p-value < 0.05 was considered statistically significant.

The primary outcome of successful abortion was achieved significantly more frequently in the study group than in the control group (80.5% vs. 48.3%, p < 0.001) (Figure 1).

Women in the control group more often required additional interventions, including a second course of misoprostol (43.3% vs. 13.6%, p = 0.001) and a third misoprostol course or alternative abortion modality such as intrauterine balloon catheter placement, oxytocin infusion, or surgical evacuation (8.3% vs. 2.5%, p = 0.078). Among women who achieved successful abortion, no statistically significant difference was observed in the induction-to-expulsion interval between groups. The mean time from the first dose of misoprostol to fetal expulsion was 586.2 ± 309.0 minutes (9.7 ± 5.1 hours) in the study group and 647.5 ± 307.5 minutes (10.7 ± 5.1 hours) in the control group (p = 0.158). Similarly, the mean interval from the last dose of misoprostol to fetal expulsion was 242.2 ± 315.5 minutes (4.0 ± 5.2 hours) in the study group and 277.9 ± 296.8 minutes (4.6 ± 4.9 hours) in the control group (p = 0.276). 

Overall complications were significantly less frequent in the study group compared with the control group (1.7% vs. 10.0%, p = 0.031). Profuse bleeding, defined as a hemoglobin decline greater than 25% from baseline, occurred in 0.8% of women in the study group compared with 8.3% in the control group (p = 0.028). The mean percentage decline in hemoglobin was also significantly lower in the study group than in the control group (8% vs. 11%, p = 0.045). There were no statistically significant differences between groups in infection rates or fever ≥38°C.

Analgesic requirements differed significantly between groups. Women in the study group required less intravenous paracetamol than those in the control group (mean 1.15 g vs. 1.64 g, p = 0.011). Similarly, opioid analgesic use, measured as intravenous pethidine hydrochloride, was significantly lower in the study group (mean 75 mg vs. 225 mg, p = 0.028). Use of other non-opioid analgesics did not differ significantly between groups (Table 2).

**Table 2:** Comparison of analgesic requirements between groups Data are presented as mean ± SD. Analgesic consumption was compared using the Mann-Whitney U test, with test statistics reported as Z-values. A p-value < 0.05 was considered statistically significant. SD, standard deviation.

Analgesic	Misoprostol (mean ± SD)	Mifepristone-misoprostol (mean ± SD)	p-value Mann-Whitney (p<0.05)	V-value
Paracetamol (g)	1.64 ± 1.29	1.15 ± 0.61	0.011	Z = −2.55
Metamizole (mg)	1514 ± 895	1347 ± 843	0.20	Z = −1.28
Diclofenac (mg)	100 ± 61	84 ± 25	0.46	Z = −0.74
Ibuprofen (mg)	523 ± 252	446 ± 173	0.19	Z = −1.31
Lornoxicam (mg)	0	8 ± 0	—	
Tramadol (mg)	100	100	1.00	Z = 0.00
Pethidine (mg)	225 ± 203	75 ± 0	0.028	Z = −2.20

Multivariable logistic regression analysis demonstrated that both treatment protocol and obstetric history were independently associated with abortion success. Use of the mifepristone-misoprostol regimen was associated with a significantly higher likelihood of successful abortion compared with misoprostol alone (odds ratio (OR): 4.6, 95% confidence interval (CI): 2.11-10.04, p < 0.001). In addition, each prior delivery increased the likelihood of success (OR: 1.4, 95% CI: 1.04-1.88, p < 0.05). Maternal age and gestational age were not independently associated with abortion success.

## Discussion

This study compared the effectiveness and safety of two protocols for second-trimester pregnancy termination. A combination regimen of mifepristone (600 mg) followed by misoprostol (400 μg) was given at a short interval (12-24 hours), and a misoprostol-only regimen. Our results show that the short-interval mifepristone-misoprostol combination significantly increases the success rate of abortion and decreases complications compared with misoprostol alone. Although the combination regimen was associated with a numerically shorter induction-to-expulsion interval, this difference was not statistically significant. These findings support using the short-interval mifepristone-misoprostol protocol as an effective and safe option for second-trimester medical abortion.

A significant difference in success rates was observed between the two treatment protocols. Women who received mifepristone followed by misoprostol had a much higher success rate than those treated with misoprostol alone (80.5% vs. 48.3%, p < 0.001). These results are consistent with previous studies showing the superiority of combination therapy, as mifepristone helps ripen the cervix and makes the uterus more sensitive to prostaglandins, which improves uterine contractions and overall abortion success [7,8,10]. Notably, many earlier studies used longer intervals between mifepristone and misoprostol, whereas our findings suggest that similar success can be achieved with a shorter 12-24-hour interval.

The optimal interval between mifepristone and the start of misoprostol remains debated, with regimens ranging from ≤12 hours to 48 hours. In this study, the short 12-24-hour interval was linked to a significantly higher chance of completing the abortion with just one dose of misoprostol, without compromising safety. This suggests that the main benefit of the short-interval regimen is increasing the chances of success, rather than speeding up the expulsion process. These results match previous research showing that shorter intervals are practical and effective [2,7,8,10]. Among women who successfully expelled the fetus during the first misoprostol dose, the average time from the first dose to fetal expulsion was shorter in the combination group compared with the misoprostol-only group; however, the difference was small and not statistically significant. This highlights the importance of cautious interpretation of time-related outcomes and indicates that the main advantage of adding mifepristone is improved effectiveness, not necessarily a shorter induction time.

The mifepristone-misoprostol regimen also had a better safety profile. The overall complication rate was significantly lower in the combination group (1.7% vs. 10.0%, p = 0.031). Bleeding was significantly more common in the misoprostol-only group, consistent with prior reports showing less blood loss with combination therapy [17]. Additionally, the decline in hemoglobin was smaller in the combination group, suggesting better bleeding control, possibly due to improved cervical preparation and more coordinated uterine contractions after mifepristone. Pain management was also better in the combination group, with lower use of intravenous paracetamol and opioids, suggesting less discomfort during the procedure. Although pain perception varies, these findings are in line with earlier research showing reduced analgesic needs with combination regimens. A prospective study by Kapp et al. also found lower opioid requirements among women given mifepristone before misoprostol in the second trimester [18].

Prior obstetric history was an important predictor of success. Each previous delivery increased the likelihood of a successful abortion, and using the mifepristone-misoprostol protocol was independently linked to more than a fourfold increase in success. These results support previous observations that prior pregnancies may improve uterine responsiveness, possibly through physiological and mechanical cervical factors. In contrast, maternal age and gestational age were not independently associated with success, consistent with earlier studies suggesting these factors have a limited impact on second-trimester medical abortion outcomes.

Several aspects of this study merit discussion in relation to existing literature. Most contemporary studies evaluating short-interval regimens have used a 200 mg dose of mifepristone, whereas our study employed a 600 mg dose, reflecting the standard institutional protocol during the study period and earlier guideline recommendations. While our results demonstrate excellent efficacy and safety with this regimen, it remains unclear whether similar outcomes could be achieved with lower doses, which may be more cost-effective and are now commonly recommended. This question warrants further investigation in prospective comparative studies and should be considered when interpreting our findings in the context of current practice.

A key strength of our study is the broad gestational age range (13-23 weeks), unlike prior studies that included only 18-23 weeks or other cut-offs [5,8,18]. This allows for a more comprehensive evaluation of the combination regimen across the second trimester. Moreover, our study is among the few that evaluate shorter intervals (12-24 hours) between mifepristone and misoprostol, providing novel insights into optimizing abortion protocols.

However, this study has several limitations. Its retrospective design introduces potential sources of bias, including unmeasured confounding and temporal changes in clinical practice. Although treatment protocols were applied according to predefined time periods rather than physician preference, residual confounding related to changes in clinical management over time cannot be fully excluded. In addition, while the study period predates some recent guideline updates, the clinical question addressed remains relevant, particularly given ongoing interest in optimizing mifepristone-misoprostol intervals and minimizing treatment burden. The study population consisted exclusively of women with confirmed intrauterine fetal demise, which may limit generalizability to second-trimester terminations performed for other indications, such as fetal anomalies or maternal health conditions. The physiological context of fetal demise may differ from other indications, potentially affecting uterine responsiveness and clinical outcomes. This distinction should be taken into account when extrapolating our results to broader populations.

## Conclusions

In conclusion, our findings indicate that a short-interval mifepristone-misoprostol regimen is associated with significantly higher success rates and fewer complications compared to misoprostol alone in the management of second-trimester intrauterine fetal demise. While the addition of mifepristone did not significantly shorten induction-to-expulsion time among successful cases, it substantially reduced the need for additional interventions and analgesia. These results support the use of short-interval combination therapy as an effective and safe option and highlight the need for future prospective studies to further refine optimal dosing and timing strategies.

## References

[1] Lohr PA, Hayes JL, Gemzell-Danielsson K (2008). Surgical versus medical methods for second trimester induced abortion. Cochrane Database Syst Rev.

[2] Henkel A, Lerma K, Blumenthal PD, Shaw KA (2020). Evaluation of shorter mifepristone to misoprostol intervals for second trimester medical abortion: a retrospective cohort study. Contraception.

[3] Arey W, Lerma K, Carpenter E (2023). Abortion access and medically complex pregnancies before and after Texas Senate Bill 8. Obstet Gynecol.

[4] Lerma K, Shaw KA (2017). Update on second trimester medical abortion. Curr Opin Obstet Gynecol.

[5] Kelly T, Suddes J, Howel D, Hewison J, Robson S (2010). Comparing medical versus surgical termination of pregnancy at 13-20 weeks of gestation: a randomised controlled trial. BJOG.

[6] Lal AK, Kominiarek MA, Sprawka NM (2014). Induction of labor compared to dilation and evacuation for postmortem analysis. Prenat Diagn.

[7] Committee on Practice Bulletins—Gynecology (2013). Practice bulletin no. 135: second-trimester abortion. Obstet Gynecol.

[8] Shaw KA, Topp NJ, Shaw JG, Blumenthal PD (2013). Mifepristone-misoprostol dosing interval and effect on induction abortion times: a systematic review. Obstet Gynecol.

[9] Allanson ER, Copson S, Spilsbury K (2021). Pretreatment with mifepristone compared with misoprostol alone for delivery after fetal death between 14 and 28 weeks of gestation: a randomized controlled trial. Obstet Gynecol.

[10] Dabash R, Chelli H, Hajri S, Shochet T, Raghavan S, Winikoff B (2015). A double-blind randomized controlled trial of mifepristone or placebo before buccal misoprostol for abortion at 14-21 weeks of pregnancy. Int J Gynaecol Obstet.

[11] Rosser S, Sekar R, Laporte J (2022). Late termination of pregnancy at a major Queensland tertiary hospital, 2010-2020. Med J Aust.

[12] Costescu D, Guilbert É (2018). No. 360-induced abortion: surgical abortion and second trimester medical methods. J Obstet Gynaecol Can.

[13] Royal College of Obstetricians and Gynaecologists (2022). Best practice in abortion care. RCOG.

[14] (2022). Abortion care guideline. WHO.

[15] Zwerling B, Edelman A, Jackson A, Burke A, Prabhu M (2024). Society of family planning clinical recommendation: medication abortion between 14 0/7 and 27 6/7 weeks of gestation: jointly developed with the Society for Maternal-Fetal Medicine. Contraception.

[16] Nagaria T, Sirmor N (2011). Misoprostol vs mifepristone and misoprostol in second trimester termination of pregnancy. J Obstet Gynaecol India.

[17] Ohannessian A, Baumstarck K, Maruani J, Cohen-Solal E, Auquier P, Agostini A (2016). Mifepristone and misoprostol for cervical ripening in surgical abortion between 12 and 14 weeks of gestation: a randomized controlled trial. Eur J Obstet Gynecol Reprod Biol.

[18] Kapp N, Borgatta L, Stubblefield P, Vragovic O, Moreno N (2007). Mifepristone in second-trimester medical abortion: a randomized controlled trial. Obstet Gynecol.

